# The Effects of an Inclusive Badminton Program on Static Postural Control for Individuals with Intellectual and Developmental Disabilities

**DOI:** 10.3390/ijerph21020210

**Published:** 2024-02-10

**Authors:** Alana J. Turner, Harish Chander, Sachini N. K. Kodithuwakku Arachchige, Aaron Griffith, Po-Lin Chen, Chih-Chia (JJ) Chen, Adam C. Knight, Kayla Bates-Brantley, Kasee Stratton-Gadke, J. Chadwick Smith

**Affiliations:** 1 Department of Kinesiology, Coastal Carolina University, Conway, SC 29626, USA; jsmith6@coastal.edu; 2 Department of Kinesiology, Mississippi State University, Mississippi State, MS 39762, USAag2843@msstate.edu (A.G.); pc918@msstate.edu (P.-L.C.); aknight@colled.msstate.edu (A.C.K.); 3 Department of Exercise and Nutrition Science, Weber State University, Ogden, UT 84403, USA; skodi@weber.edu; 4 Department of Counseling, Educational Psychology, and Foundations, Mississippi State University, Mississippi State, MS 39762, USA; keb240@msstate.edu (K.B.-B.);

**Keywords:** exercise intervention, disabilities, adapted physical activity

## Abstract

The purpose of the study was to examine static postural control/balance in young adults with intellectual and developmental disabilities (IDD) and typically developing (TD) young adults before, during, and after an inclusive badminton intervention. Eight participants (four IDD-BADM and four TD-BADM) participated in a 12-week inclusive badminton intervention, with the other eight participants as matched controls (four IDD-CONTR and four TD-CONTR) (74.19 kg ± 9.8 kg, 171.96 cm ± 5.4 cm; 21.7 ± 1.8 years of age; nine females and seven males; eight with IDD and eight TD). The study followed a repeated measures design (pre, mid, post) before the intervention, at 6 weeks, and after 12 weeks. Static postural sway conditions included: bilateral stance eyes open (20 s), eyes closed (10 s), foam eyes open (20 s), foam eyes closed (10 s), and unilateral stance eyes open (10 s) and foam eyes open (10 s). Sway measurements included: average anterior/posterior (A/P) displacement (in), average medial/lateral (M/L) displacement (in), average 95% ellipsoid area (in^2^), and average velocity (ft/s). Significant time × group interactions were reported for average velocity (EO) (*p* = 0.030), average length (EO) (*p* = 0.030), 95% ellipsoid area (EO) (*p* = 0.049), and average A/P displacement (1LEO) (*p* = 0.036) for IDD-BADM. Significant time main effects were reported for average A/P displacement (FEO) (*p* = 0.040) for IDD groups. Significant time main effects were reported for average M/L displacement (EO) (*p* = 0.001), (EC) (*p* = 0.004), (FEO) (*p* = 0.005), (FEC) (*p* = 0.004), and average A/P displacement (EO) (*p* = 0.006) and (FEO) (*p* = 0.005) for TD groups. An inclusive badminton program indicated evidence of improved static postural control for those with IDD. However, no significant differences were reported for TD peers.

## 1. Introduction

Within the general population, 1–3% of individuals are diagnosed with intellectual and developmental disabilities (IDD) [1,2,3]. According to the American Psychiatric Association and the American Association on Intellectual Developmental Disabilities, IDD is characterized by two areas of functioning, “intellectual functioning (learning, problem-solving, judgment) and adaptive functioning (activities of daily living (ADL) like communication and independent living)” [4,5]. Adaptive functioning is also subcategorized into three areas: conceptual (reasoning and knowledge memory), social (communication and social judgment), and practical (independent living, organizing work tasks, and recreation) [5,6]. With health and mobility, all three areas of adaptive functioning are impacted for those with IDD when compared to their typically developing (TD) peers. Moreover, individuals with IDD experience a multitude of health disparities including increased risks of diabetes, obesity, hypertension, high cholesterol, and deficits in overall movement and postural control mechanisms such as static postural control [7]. For example, individuals with IDD present an array of postural control and locomotor deficits which results in a higher prevalence of falls when compared to TD peers [2]. Previous literature has reported that the fall prevalence for adults with IDD is over 40% [2]. Thirty-two percent of these falls result in injury or death [2]. Therefore, efforts to improve postural control mechanisms for individuals with IDD could be life-altering. 

Postural control is the ability to maintain postural equilibrium and orientation by constant adjustment of an individual’s center of gravity (COG) within their base of support (BOS) [8]. When there are deficits in the postural control system, overall movement and limb coordination tend to be impacted. Sensorimotor issues, such as decreases in postural control [9,10,11], poor limb coordination [12], gait abnormalities [13,14], and reduced anticipatory postural adjustments of motor actions [15,16] are frequently reported in individuals with developmental disabilities which could be considered symptoms supporting a diagnosis of IDD like ASD [5]. Higher-level processes of motor control mechanisms are utilized to maintain postural equilibrium and coordinate movement such as sensory integration, sensory organization, and feedforward and feedback processes. Continued control of motor behaviors includes the coordination of several joints, simultaneously. The coordination of the body as a combination of multi-joint segments allows the individual to initiate complex movements like grasping, reaching, and adjusting the balance utilized for an individual’s gait [17]. Also, feedforward processes ensure the individual can maintain control of fluid motor behaviors since they are involved in preparing and executing quick movements based on the sensory feedback readily available from the environment [17,18]. To maintain or improve the above-mentioned postural control mechanisms, training is typically required, especially for those with IDD. Previous literature reported that physical activity can improve static postural control, e.g., creative dance, balance exercises, Wii Fit, and Tai Chi [19,20,21,22]. However, individuals with IDD tend to have limited access to unique training opportunities when compared to their TD peers, especially adapted sports. Therefore, the current study aimed to evaluate an inclusive adapted physical activity, badminton, on postural control in individuals with IDD. 

Badminton is a popular sport worldwide that requires fast, powerful shots and agile footwork [23]. Badminton players must react to the moving shuttlecock and adjust their body position continuously throughout the game [20]. They must maintain their COG within their BOS while performing very rapid and asymmetrical upper limb movements [24]. Therefore, superior balance is crucial for badminton skill advancement and sports performance [24]. Previous literature reported that when standing on the non-dominant leg with their eyes closed, badminton players’ postural sway decreased over time [25]. Furthermore, previous research revealed that 8 weeks of badminton training can improve dynamic functional balance performance in TD children [25]. Agility-type footwork such as the ability to alter direction over short distances is essential in both defending and attacking maneuvers during badminton training and competitions [26,27,28,29]. Agility, which is defined as a rapid whole-body movement with a change in the magnitude of velocity or direction in response to a stimulus [26] is a crucial variable for outstanding performance in badminton competitions [27,28,29]. Therefore, agility-type training during badminton could not only improve balance for young adults with IDD but could improve postural control mechanisms for this population. However, postural control mechanisms have not yet been fully examined for young adults with IDD after an inclusive badminton intervention. Therefore, the purpose of this study was to evaluate the implementation of an inclusive badminton program as an adapted physical activity for young adults with IDD to improve areas of postural control. 

## 2. Materials and Methods

### 2.1. Participants

Sixteen male and female participants (74.19 kg ± 9.8 kg, 171.96 cm ± 5.4 cm; 21.7 ± 1.8 years of age; nine females and seven males; eight with IDD and eight with TD) started and completed the study; therefore, no participants were released or discontinued the study. Participants met the following inclusion criteria: (1) being between the ages of 18 and 30; (2) (a) participants with IDD specifically were students in a comprehensive transition program for intellectual disabilities at a southeastern university (b) TD participants were also recruited from the same southeastern university. Participants with IDD were specifically recruited from the abovementioned program and selected for the badminton intervention based on a randomization selection. Participants were randomized and placed into four groups: two groups participating in the badminton intervention (four students with IDD (IDD-BADM) and four TD participants (TD-BADM)) and two control groups not participating in the badminton intervention four students with IDD (IDD-CONTR) and four TD participants (TD-CONTR)). Controls for IDD were selected and matched based on diagnosis. The four IDD-BADM participants included diagnoses of high-functioning ASD (two participants), DS (one participant), and hemiplegic cerebral palsy (one participant), each with a secondary diagnosis of intellectual disability (ID). Badminton participants had not previously participated in a badminton program and were asked to not participate in balance-focused training exercises such as yoga, Tai Chi, or strength-training programs during the intervention phases. Controls were instructed to continue with typical daily activities aside from a badminton program or class. 

### 2.2. Experimental Procedures 

**Scheme 1:**
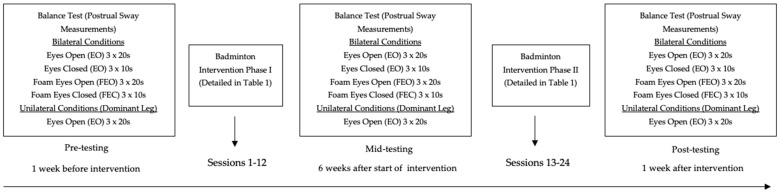
Study design.

After obtaining consent, familiarization of the study included a Par-Q+ (Physical Activity Questionnaire Plus) to ensure participants were ready for exercise along with a collection of anthropometric data. A Par-Q+ is an extensive physical activity readiness questionnaire that asks specific questions about chronic health conditions such as musculoskeletal (arthritis, rheumatoid arthritis), cardiopulmonary issues (heart conditions), hypertension, and diabetes. A within-subjects repeated-measure design was utilized for those with IDD and TD which included three testing days (pre-test, mid-test, and post-test) within a 12-week badminton-adapted physical education class as a physical activity intervention. The study took place across 14 weeks (August–December 2021) including one week each of pre-testing and post-testing. The pre-tests occurred one week before the badminton intervention. Mid-tests were 6 weeks after the start of the intervention, while post-testing followed one week after the intervention. All testing days included static balance tests where participants stood on an AMTI^®^ force plate platform (Waterton, MA, USA) and Airex AG^®^ balance pad (L: 48 cm × W: 40 cm × H: 6 cm) (for foam conditions) under the following conditions: Bilateral stance: eyes open (EO)-20 s, eyes closed (EC)-10 s, foam eyes open (FEO)-20 s, foam eyes closed (FEC)-10 s and unilateral stance on participant’s dominant leg with the eyes open (1LEO)-10 s. Participants were instructed to keep their eyes ahead and arms by their sides. Leg dominance was determined by the preferred leg to kick a ball. All testing took place in a controlled, distraction-less laboratory setting to not impact balance measurements while the badminton class was held in a gymnasium setting with minimum distractions. The adapted physical education class followed the Special Olympics Individual Badminton Skills Assessment, the Badminton World Federation (BWF) guidelines (Table 1) and was designed as a bi-weekly 50-min badminton adapted physical education class. The class structure included 5 min of dynamic warm-up, 40 min of badminton instruction (Table 2) by a Certified Adapted Physical Education (CAPE) instructor and two graduate teaching assistants for 12 weeks (24 sessions), and a 5-min cool-down of static stretching. TD-BADM participants were more involved in instructing the participants with IDD rather than participating in the badminton drills themselves towards the end of the intervention (sessions 16–24); however, TD-BADM were involved in “game play” at the end of each session. All badminton participants were instructed to not participate in any balance training programs like yoga or Tai Chi throughout the intervention. Scheme 1 depicts the visual representation of the experimental procedures.

### 2.3. Data Analysis

Analyses of center of pressure (COP) measurements were derived from the force platform to quantify postural sway as a measure of postural stability. Postural sway variables included average displacements in the medial–lateral (M/L) and anterior–posterior (A/P) directions (in.), average 95% ellipsoid area (in^2^), and average velocity (ft/s), and average length (in). All postural sway dependent variables were calculated for the three testing time periods (pre, mid, post) and two group types ((IDD–BADM, IDD–CONTR) (TD-BADM, TD–CONTR)) during all six static balance conditions (EO, EC, EOF, ECF, 1LEO). All the study variables are listed in Table 3. 

### 2.4. Statistical Analysis

The dependent COP postural sway variables were analyzed using a between-subjects 2 × 3 (group × time) repeated measures analysis of variance [2 (IDD-BADM × IDD-CONTR) × 3 (Pre–test × Mid-test × Post–test)] repeated measures analysis of variance (RM ANOVA) and 2 × 3 (group × time) repeated measures analysis of variance [2 (TD-BADM × TD-CONTR) × 3 (Pre–test × Mid–test × Post–test)] (RM ANOVA) independently utilizing a Greenhouse–Geisser correction. Post-hoc pairwise comparisons were performed with a Bonferroni correction if significant main effects were identified. All statistical analyses were performed using JASP (Version 0.18.1 (Intel)) (Amsterdam, The Netherlands) at alpha level *p* ≤ 0.05. All statistical significance is listed in Table 4 as a summarized table. 

## 3. Results

Participants involved in the badminton program were placed into two separate groups (IDD-BADM) and (TD-BADM) and matched with the assigned control groups that did not partake in the badminton intervention (IDD-CONTR) and (TD-CONTR). All groups completed three testing periods over 12 weeks of the inclusive badminton intervention: pre-testing (1 week before intervention), mid-testing (6 weeks during intervention), and post-testing (1 week after intervention) under the following conditions: bilateral stance EO, EC, FEO, FEC, and unilateral stance on the dominant leg 1LEO. Significant main results are listed for each group under these conditions. All repeated measures ANOVA results are listed in Table 4 while means and standard deviations are listed in Table 5.

### 3.1. Eyes Open (EO)

**Figure 1:**
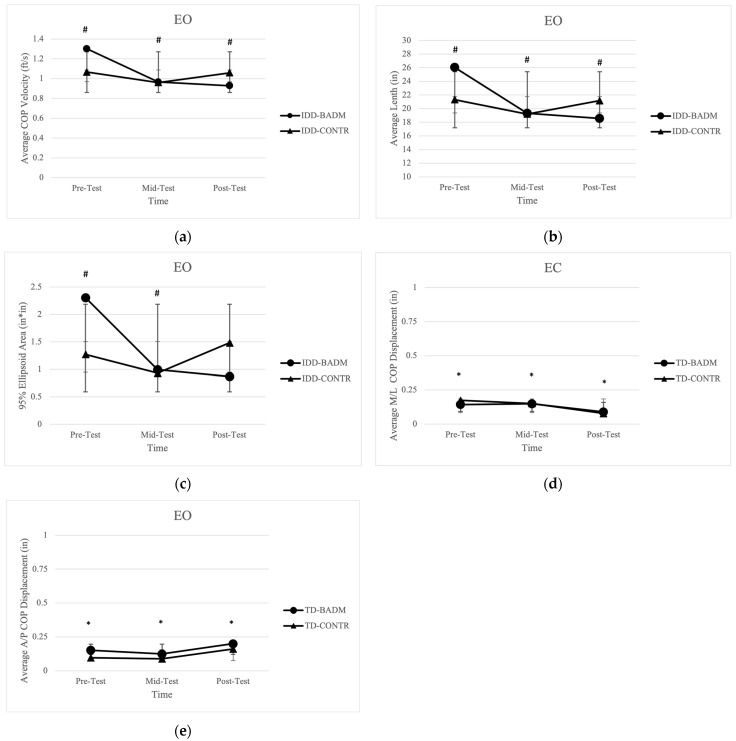
(**a**) Average COP velocity IDD groups. “#” indicates significance (group × time interaction). (**b**) Average length for IDD groups. “#” indicates significance (group × time interaction). (**c**) 95% ellipsoid area for IDD groups. “#” indicates significance (group × time interaction). (**d**) Average M/L COP displacement for TD groups. “*” indicates significance (time-main effect). (**e**) Average A/P COP displacement for TD groups. “*” indicates significance (time-main effect).

Significant time × group interactions were shown for IDD-BADM for average velocity [F (2, 12) = 7.415, (*p* = 0.030), (η_p_^2^ = 0.673)] with pairwise comparisons from pre-mid (*p* = 0.002) and pre-post (*p* = 0.006) (Figure 1a); average length [F (2, 12)= 7.442, (*p* = 0.030), (η_p_^2^ = 0.533)] with pairwise comparisons pre-mid (*p* = 0.002) and pre-post (*p* = 0.006) (Figure 1b), and 95% ellipsoid area [F (2, 12) = 3.934, (*p* = 0.049), (η_p_^2^ = 0.396)] with significant pairwise comparisons from pre-mid testing (*p* = 0.048) (Figure 1c), indicating lower balance scores for IDD-BADM when comparing pre-mid testing and pre-post testing for all variables. Average velocity, average length, and 95% ellipsoid area decreased for both IDD groups from pre-mid and pre-post testing; however, from pre-post testing IDD-BADM continued to decrease COP velocity and length while IDD-CONT increased COP velocity over the twelve weeks, resulting in decreases in balance performance for the control group. Similar results were reported for ellipsoid areas with decreases in balance scores from pre-mid testing where IDD-BADM continued to improve their balance performance compared to the CONTR group. No significant time or group main effects, nor interactions were found for average displacement in the M/L or A/P directions. 

Significant time main effects were reported for both TD-BADM and TD-CONTR for average COP displacement in the M/L direction [F (2, 12)= 15.814, (*p* = 0.001), (η_p_^2^ = 0.725)] with pairwise comparisons pre-post (*p* = 0.005) and mid-post (*p* = 0.001) (Figure 1d) and the average displacement in the A/P direction [F (2, 12)= 10.494, (*p* = 0.006), (η_p_^2^ = 0.636)] pre-post (*p* = 0.017) and mid-post (*p* = 0.003) (Figure 1e). Both TD groups exhibited improvements in balance performance in the M/L direction from pre-mid testing and overall pre-post testing. Yet, both TD groups revealed decreases in balance performance from pre-mid testing and pre-post testing for the A/P direction. No significant time or group main effects, nor interactions were found for 95% ellipsoid area, average velocity, or average length. 

### 3.2. Eyes Closed

**Figure 2:**
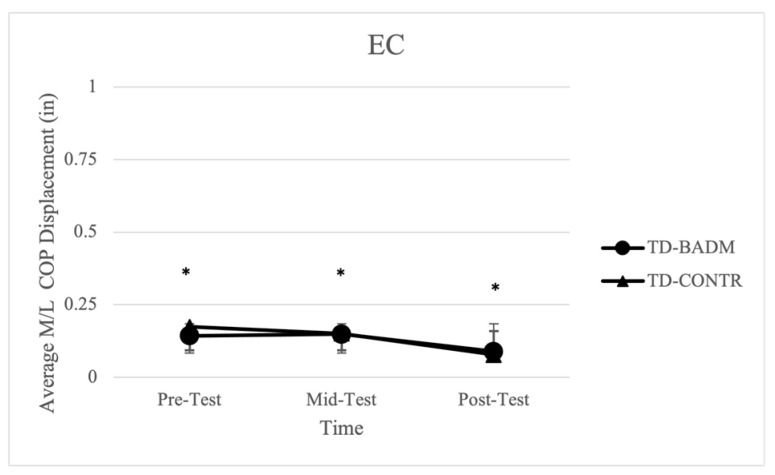
Average M/L COP displacement for TD groups (EC). “*” indicates significance (time-main effect).

Significant time-main effects were reported for both TD-BADM and TD-CONTR in average displacement in the M/L direction [F (2, 12) = 12.146, (*p* = 0.004), (η_p_^2^ = 0.669)] with significant pairwise comparisons from pre-post (*p* = 0.002) and mid-post (*p* = 0.005) (Figure 2). Both TD groups decreased M/L displacement from pre-mid testing and pre-post testing, resulting in an improvement in balance measurements. No time or group main effects nor interactions were found for the 95% ellipsoid area, average velocity, or average length or in the A/P direction. Further, no significant time or group main effects, nor interactions were found for IDD-BADM or IDD-CONTR.

### 3.3. Foam Eyes Open (FEO) 

**Figure 3:**
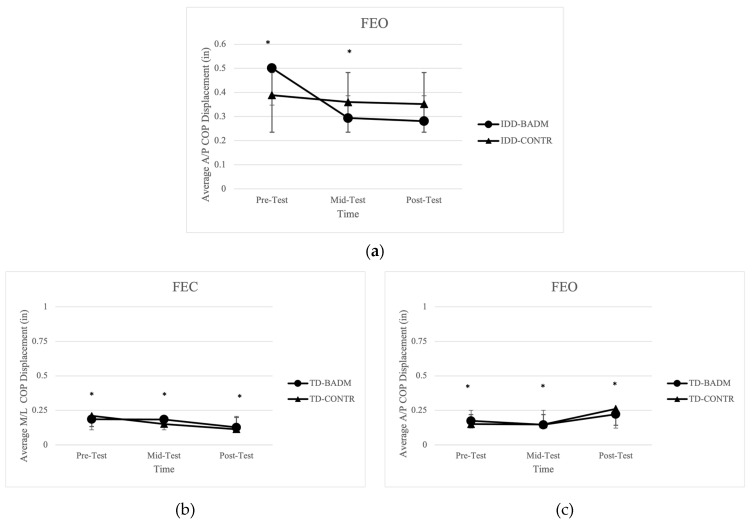
(**a**) Average A/P COP displacement for IDD groups. “*” indicates significance (time-main effect). (**b**) Average M/L COP displacement for TD groups. “*” indicates significance (time-main effect). (**c**) Average A/P COP displacement for TD groups. “*” indicates significance (time-main effect).

Significant time main effects were present for IDD-BADM and IDD-CONTR in average displacement in the A/P direction [F (2, 12) = 5.708, (*p* = 0.040), (η_p_^2^ = 0.488)] with a significant pairwise comparison from pre-post (*p* = 0.031) and pre-mid (*p* = 0.048), demonstrating decreasing postural sway from pre-mid testing (Figure 3a). No significant time or group main effects, nor interactions were found for average displacement in the M/L, 95% ellipsoid area, average velocity, or average length.

Significant time main effects were reported for TD-BADM and TD-CONTR in average displacement in the M/L direction [F (2, 12)= 11.416, (*p* = 0.005), (η_p_^2^ = 0.530)] with significant pairwise comparisons from pre-post (*p* = 0.003) and mid-post (*p* = 0.007) (Figure 3b) and average displacement in the A/P direction [F (2, 13.709), (*p* = 0.001), (η_p_^2^ = 0.696)] (Figure 3c) with significant pairwise comparisons from pre to post (*p* = 0.005) and mid-post (*p* = 0.001), demonstrating decreases in M/L displacement from pre-post testing and mid-post testing; however, A/P displacement increased from pre-post testing and mid-post testing. No significant time or group main effects, nor interactions were found for 95% ellipsoid area, average velocity, or average length. 

### 3.4. Foam Eyes Closed (FEC)

**Figure 4:**
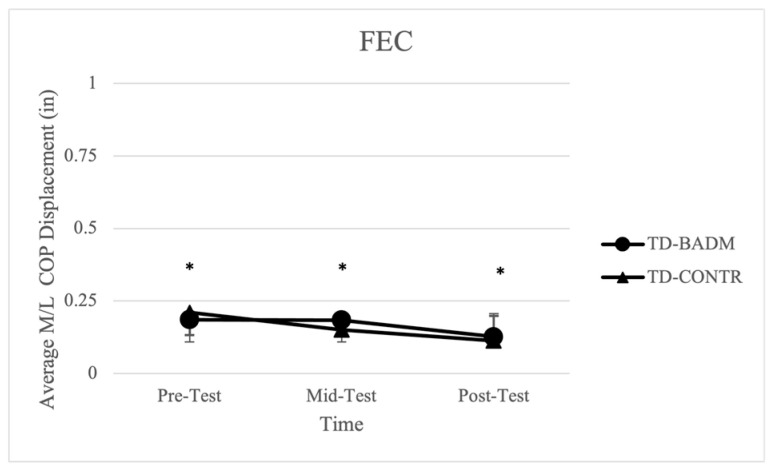
Average M/L COP displacement for TD groups (FEC). “*” indicates significance (time-main effect).

Significant time main effects were found for TD-BADM and TD-CONTR in average displacement in the M/L direction [F (2, 12) = 9.489, (*p* = 0.004), (η_p_^2^ = 0.613)] with significant pairwise comparisons from pre-post (*p* = 0.003), which exhibited decreases in displacement revealing improvements in balance measures (Figure 4). For IDD-BADM and IDD-CONTR, no significant time or group main effects, nor interactions were found for average displacement in the M/L or A/P directions, 95% ellipsoid area, average velocity, or average length. 

### 3.5. Unilateral Eyes Open (1LEO)

**Figure 5:**
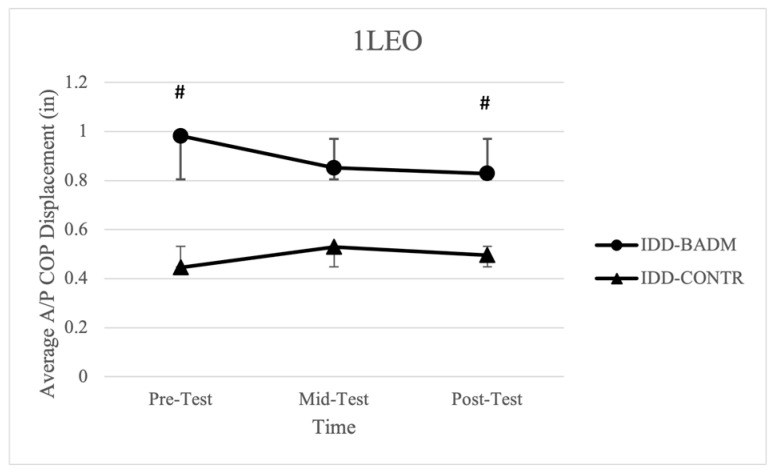
Average A/P COP displacement for IDD groups. “#” indicates significance (group × time interaction).

A significant time × group interaction was reported for IDD-BADM in average displacement in the A/P direction [F (2, 12) = 4.621, (*p* = 0.036), (η_p_^2^ = 0.435)] with a significant pairwise comparison from pre-post (*p* = 0.022) (Figure 5). IDD-BADM had significantly less A/P displacement from pre-post testing when compared to the control group, showing improvements in postural sway variables over time for the intervention group. However, no significant time or group main effects, nor interactions were found for average displacement in the M/L direction, 95% ellipsoid area, average velocity, or average length. IDD-CONTR, TD-BADM, and TD-CONTR reported no significant time or group main effects, nor interactions were found for average displacement in the A/P direction, 95% ellipsoid area, average velocity, or average length.

## 4. Discussion

This study evaluated the effects of a 12-week inclusive badminton intervention on postural control in young adults with IDD. Through this program, the researchers attempted to develop an adapted, inclusive physical activity program to improve postural control. The results of this study suggest that badminton may be an effective balance training alternative among young adults with IDD. However, for TD peers, postural control was maintained across the 12 weeks, and no significant group differences were observed for TD-BADM when compared to TD-CONTR.

Average postural sway variables included: average COP A/P displacement, average COP M/L displacement, 95% ellipsoid area, average COP velocity, and average COP length under the following conditions EO, EC, FEO, FEC, 1LEO. Significant time × group interactions for IDD-BADM were reported during EO and 1LEO, exhibiting considerably lower sway measurements in the young adults with IDD who participated in the badminton intervention. Significant time main effects were observed for the IDD groups during FEO and TD groups during EO, EC, FEO, and FEC. 

For the EO condition, significant time × group interactions for average velocity, length, and 95% ellipsoid area were reported for the IDD groups with decreases in static and bilateral stance. IDD-BADM had a decrease in average velocity, average length, and 95% ellipsoid area for the EO condition at 6 weeks and continued to have velocity and length decreases after 12 weeks of training compared to IDD-CONTR. IDD-BADM exhibited greater decreases in COP sway variables for average velocity, average length, and 95% ellipsoid area when compared to their matched controls. Various types of balance training intervention studies stated similar decreases in COP sway variables from strength and balance training [20], Wii Fit^®^ balance game training [21], and Tai Chi [22]. Badminton and similar adapted balance interventions suggest activities that focus on balance training characteristics (weight shifts, unilateral stance, limits of stability testing) could improve postural control mechanisms. 

During the EO condition, significant time main effects in average COP M/L and A/P displacement for both TD groups were reported. While COP M/L displacement decreased over the 12 weeks for both groups, average COP A/P displacement during EO and FEO increased for both groups after 12 weeks, which reveals some balance degradation throughout the intervention for badminton participants and controls. These findings for average COP A/P displacement decrements could be related to the student development aspect of the class for TD-BADM. For example, shortly after six weeks into the intervention (sessions 16–24), TD-BADM began to correct the IDD-BADM participants during the adapted physical education class instead of participating in the drills provided by the graduate students. Due to the nature of the corrections, the participants were unable to actively participate in the badminton drills to improve their own skills. Moreover, TD-BADM still participated in the “game play” portion of the class structure. However, this may have impacted the TD-BADM results for the post-testing and is a possible limitation of the study. In addition, the time frame of the data collection of the post-tests could have affected the results. Upon entry into the laboratory, participants reported how they were feeling based on a 5-point pictorial Likert scale. Participants reported higher level scores after the 12 weeks than in pre-testing. The higher-level scores could be correlated with tiredness and fatigue due to emotional stress from external factors like final examinations and lack of sleep which justifies the possible higher sway increases in the A/P direction for the TD participants and controls. Future studies utilizing a similar intervention should carefully track if these measures also show a degradation of postural control mechanisms over time. 

During EC, FEO, and FEC, both TD groups showed decreases in average M/L displacement from pre-post and mid-post testing as significant time main effects. Also, in the FEO condition, both IDD groups decreased the average sway A/P direction from pre-mid and pre-post testing. For TD-BADM and IDD-BADM, these results are like previous studies and could be related to the type of progression through the 12 weeks of the intervention as the skills intensified from the static position underhand serve to more dynamic movements such as a forehand and backhand, focusing on weight shifts in the medial and lateral directions and agility [27,28,29]. However, we could not control for TD-CONTR, so their improvements could be related to their own physical activity throughout the 12 weeks. 

For 1LEO, IDD-BADM reported a significant time × group interaction for average A/P displacement from pre-post testing, decreasing the A/P displacement of their COP when compared to IDD-CONTR after the 12-week intervention. These results align with the type of movements and skills that are acquired from playing badminton. For instance, badminton players react to the moving shuttlecock and adjust their body position rapidly and accordingly throughout the game [27]. Badminton players are repetitively shifting their COG outside their BOS while performing very quick, unilateral upper limb movements [23]. This constant movement of the COG with asymmetrical upper body movements while playing badminton over the course of the 12-week biweekly classes is challenging, and trains the postural control system by integrating and organizing changing sensory information while utilizing a feedforward process for quick response times, especially for those with postural control deficits. This type of intervention is also training anticipatory postural adjustments which assist in the A/P displacement of the individual’s COG while moving forwards and backwards throughout the court and while the individual is contacting the shuttlecock with a rapid change in the six degrees of freedom of the dominant arm’s glenohumeral joint of the upper extremity. Even though these are training dynamic balance movements, static balance like during the 1LEO condition is also being challenged and improving. 

## 5. Conclusions

For young adults with IDD, postural control and balance deficits are observed and reported for this population, resulting in poor gross motor function, falls, and limited physical activity participation. Badminton could be an inclusive and adapted physical activity to improve static postural control. Results from this study demonstrate that badminton could be an efficient form of physical activity to increase postural control for those with IDD. However, this study did not find significant group differences for the TD peers when compared to controls. This could be related to the design of the class. The TD peers were more involved in teaching and instructing the participants with IDD rather than participating in the badminton drills themselves towards the end of the intervention which could be a limitation. One limitation of the study includes the small sample size of all groups, which could contribute to the results. Also, participants were instructed to not participate in balance-related activities such as yoga, strength training, and Tai Chi among other exercise activities established to improve postural control mechanisms. Further, we could not control this external factor, so it could be an added limitation. Participants from each group could have participated in other balance training programs throughout the study, even though they were instructed not to. One major strength of this research study was the inclusive nature of the intervention design. For example, it is important to improve the physical components of quality of life for those with IDD; however, there should be an equal amount of importance in promoting inclusivity, especially for exercise. Exercise adherence tends to be an area of interest for those with IDD. Inclusive activities with TD peers could be the key to maintaining and upholding an exercise regime for those with IDD. The practical implications of the study align with the need to create fall prevention programs to reduce the prevalence of falls for this population, while promoting inclusion with TD peers and physical activity participation. Creating adaptive, inclusive programs could break barriers to socialization for those with IDD while improving quality life aspects like postural control mechanisms. Traditional balance training activities could be isolating and decrease exercise adherence. Further, creating inclusive exercise programs could motivate those with IDD to not only improve balance but also to become physically active with TD peers. Future studies could explore other areas of movement, like dynamic balance, physical fitness, and the effects of badminton with larger sample sizes. In addition, future research could explore other inclusive and adapted physical activities, like badminton, with agility-like training such as tennis and pickleball. 

## Figures and Tables

**Table 1:** Badminton intervention program.

Badminton Intervention Phase I (Sessions 1–12)	Badminton Intervention Phase II (Sessions 13–24)
Grips Wrist movements Ready position Basic underhand stroke Low short serve (backhand) Serve returns Underhand net rally	High deep serves Forehand overhead clear Forehand overhead smash Overhand backhand clear Overhand backhand drop

**Table 2:** Adapted physical education class structure.

Dynamic Warm-Up (10 min)	Badminton Instruction (30 min)	Cooldown (10 min)
Walking laps (around gym) Arm circles and swings Heel walks Toe walks Walking quadriceps stretch Lunges Side Shuffles	Introduction of new skill (5 min) Practice with TD peer through drills (previous skills and new skills) (15 min) Game play (10 min)	Cross arm stretch Hamstring stretch Quadriceps stretch Rounded back stretch Butterfly stretch

**Table 3:** Study variables.

Dependent Variables	Independent Variables	Controlled Variables
COP Average displacement (A/P) direction (in.) COP Average displacement (M/L) direction (in.) COP average 95% ellipsoid area (in^2^) COP average velocity (ft/s) COP average length (in)	Groups (IDD and TD) Time of testing (Pre, Mid, Post) Balance Testing Conditions (EO, EC, FEO, FEC, 1LEO)	Groups– IDD CONTR and TD CONTR

**Table 4:** Repeated measures ANOVA table for conditions and groups.

Variable (Condition)	Main Effect or Interaction	*F*-Value	*p*-Value	η_p_^2^	Post Hoc Pairwise Comparisons and *p*-Values
Average Velocity (EO)	Time × Group Interaction (IDD-BADM)	*F* (2, 12) = 7.415	0.030	0.673	Pre > Mid *p* = 0.002 Pre > Post *p* = 0.006
Average Length (EO)	Time × Group Interaction (IDD-BADM)	*F* (2, 12) = 7.422	0.030	0.553	Pre > Mid *p* = 0.002 Pre > Post *p* = 0.006
95% Ellipsoid Area (EO)	Time × Group Interaction (IDD-BADM)	*F* (2, 12) = 3.934	0.049	0.396	Pre > Mid *p* = 0.048
Average Displacement in M/L direction (EO)	Time Main Effect (TD)	*F* (2, 12) = 15.814	0.001	0.725	Pre > Post *p* = 0.005 Mid > Post *p* = 0.001
Average Displacement in A/P direction (EO)	Time Main Effect (TD)	*F* (2, 12) = 10.494	0.006	0.636	Pre < Post *p* = 0.017 Mid < Post *p* = 0.003
Average Displacement in M/L direction (EC)	Time Main Effect (TD)	*F* (2, 12) = 12.146	0.004	0.669	Pre > Post *p* = 0.002 Mid > Post *p* = 0.005
Average Displacement in A/P direction (FEO)	Time-Main Effect (IDD)	*F* (2, 12) = 5.708	0.040	0.488	Pre > Mid *p* = 0.048 Pre > Post *p* = 0.031
Average Displacement in M/L direction (FEO)	Time-Main Effect (TD)	*F* (2, 12) = 11.416	0.005	0.530	Pre > Post *p* = 0.003 Mid > Post *p* = 0.007
Average Displacement in A/P direction (FEO)	Time-Main Effect (TD)	*F* (2, 12) = 13.709	0.005	0.696	Pre < Post *p* = 0.005 Mid < Post *p* = 0.001
Average Displacement in M/L direction (FEC)	Time-Main Effect (TD)	*F* (2, 12) = 9.489	0.004	0.613	Pre > Post *p* = 0.003
Average Displacement in A/P direction (1LEO)	Time × Group Interaction (IDD-BADM)	*F* (2, 12) = 4.621	0.036	0.435	Pre > Post *p* = 0.022

**Table 5:** Means and standard deviations.

Sway Measurement/Condition	Group	Time	Mean and SD
Average Velocity (EO)	IDD-BADM (Time × Group Interaction)	Pre-Test Mid-Test Post-Test	1.302 ± 0.451 0.966 ± 0.348 0.928 ± 0.278
Average Length (EO)	IDD-BADM (Time × Group Interaction)	Pre-Test Mid-Test Post-Test	26.039 ± 9.021 19.322 ± 6.953 18.556 ± 5.552
95% Ellipsoid Area (EO)	IDD- BADM	Pre-Test Mid-Test Post-Test	2.305 ± 1.0210.994 ± 0.560 0.869 ± 0.186
	IDD-CONTR	Pre-Test Mid-Test Post-Test	1.272 ± 0.497 0.932 ± 0.587 1.482 ± 0.066
Average Displacement in M/L direction (EO)	TD-BADM	Pre-Test Mid-Test Post-Test	0.135 ± 0.032 0.167 ± 0.051 0.113 ± 0.032
	TD-CONTR	Pre-Test Mid-Test Post-Test	0.175 ± 0.022 0.185 ± 0.018 0.058 ± 0.021
Average Displacement in M/L direction (EC)	TD-BADM	Pre-Test Mid-Test Post-Test	0.143 ± 0.044 0.149 ± 0.047 0.078 ± 0.026
	TD-CONTR	Pre-Test Mid-Test Post-Test	0.174 ± 0.043 0.151 ± 0.058 0.078 ± 0.036
Average Displacement in A/P direction (FEO)	IDD-BADM	Pre-Test Mid-Test Post-Test	0.501 ± 0.121 0.294 ± 0.075 0.281 ± 0.066
	IDD-CONTR	Pre-Test Mid-Test Post-Test	0.389 ± 0.141 0.360 ± 0.089 0.352 ± 0.035
	TD-BADM	Pre-Test Mid-Test Post-Test	0.284 ± 0.109 0.232 ± 0.037 0.139 ± 0.040
	TD-CONTR	Pre-Test Mid-Test Post-Test	0.232 ± 0.055 0.305 ± 0.059 0.133 ± 0.028
	TD-BADM	Pre-Test Mid-Test Post-Test	0.176 ± 0.029 0.146 ± 0.036 0.222 ± 0.055
	TD-CONTR	Pre-Test Mid-Test Post-Test	0.150 ± 0.019 0.148 ± 0.015 0.261 ± 0.076
Average Displacement in M/L direction (FEC)	TD-BADM	Pre-Test Mid-Test Post-Test	0.186 ± 0.019 0.184 ± 0.039 0.127 ± 0.041
	TD-CONTR	Pre-Test Mid-Test Post-Test	0.210 ± 0.050 0.151 ± 0.032 0.113 ± 0.011
Average Displacement in A/P direction (1LEO)	IDD-BADM (Time × Group Interaction)	Pre-Test Mid-Test Post-Test	0.982 ± 0.548 0.852 ± 0.589 0.829 ± 0.580

## Data Availability

The data presented in this study are available on request from the corresponding author due to privacy reasons.

## Abbreviations

Abbreviations	Definitions
COG	Center of gravity
BOS	Base of support
IDD	Intellectual and developmental disabilities
TD	Typically developing
BADM	Badminton
EO	Eyes open
EC	Eyes closed
FEO	Foam eyes closed
FEC	Foam eyes open
1LEO	Single-leg eyes open
COP	Center of pressure
CONTR	Control
